# *In Vitro* Activity of Cefotetan against ESBL-Producing Escherichia coli and Klebsiella pneumoniae Bloodstream Isolates from the MERINO Trial

**DOI:** 10.1128/spectrum.00226-21

**Published:** 2021-07-07

**Authors:** Adam G. Stewart, Kyra Cottrell, Andrew Henderson, Kanthi Vemuri, Michelle J. Bauer, David L. Paterson, Patrick N. A. Harris

**Affiliations:** a Department of Infectious Diseases, Royal Brisbane and Women’s Hospital, Brisbane, Australia; b Centre for Clinical Research, Faculty of Medicine, The University of Queensland, Royal Brisbane and Women’s Hospital Campus, Brisbane, Australia; c Central Microbiology, Pathology Queensland, Royal Brisbane and Women’s Hospital, Brisbane, Australia; d Infection Management Services, Princess Alexandra Hospital, Brisbane, QLD, Australia; Houston Methodist Hospital

**Keywords:** extended-spectrum beta-lactamase, *ampC* beta-lactamase, antimicrobial susceptibility testing, AmpC, cefotetan, *Enterobacterales*

## Abstract

Extended-spectrum-beta-lactamase (ESBL)-producing *Enterobacterales* continue to pose a major threat to human health worldwide. Given the limited therapeutic options available to treat infections caused by these pathogens, identifying additional effective antimicrobials or revisiting existing drugs is important. Ceftriaxone-resistant Escherichia coli and Klebsiella pneumoniae containing CTX-M-type ESBLs or AmpC, in addition to narrow-spectrum OXA and SHV enzymes, were selected from blood culture isolates obtained from the MERINO trial. Isolates had previously undergone whole-genome sequencing (WGS) to identify antimicrobial resistance genes. Cefotetan MICs were determined by broth microdilution (BMD) testing with a concentration range of 0.125 to 64 mg/liter; CLSI breakpoints were used for susceptibility interpretation. BMD was performed using an automated digital antibiotic dispensing platform (Tecan D300e). One hundred ten E. coli and 40 K. pneumoniae isolates were used. CTX-M-15 and CTX-M-27 were the most common beta-lactamases present; only 7 isolates had coexistent *ampC* genes. Overall, 98.7% of isolates were susceptible, with MIC_50_s and MIC_90_s of 0.25 mg/liter and 2 mg/liter (range, ≤0.125 to 64 mg/liter), respectively. MICs appeared higher among isolates with *ampC* genes present, with an MIC_50_ of 16 mg/liter, than among those containing CTX-M-15, which had an MIC_50_ of only 0.5 mg/liter. Isolates with an *ampC* gene exhibited an overall susceptibility of 85%. Presence of a narrow-spectrum OXA beta-lactamase did not appear to alter the cefotetan MIC distribution. Cefotetan demonstrated favorable *in vitro* efficacy against ESBL-producing E. coli and K. pneumoniae bloodstream isolates.

**IMPORTANCE** Carbapenem antibiotics remain the treatment of choice for severe infection due to ESBL- and AmpC-producing *Enterobacterales*. The use of carbapenems is a major driver of the emergence of carbapenem-resistant Gram-negative bacilli, which are often resistant to most available antimicrobials. Cefotetan is a cephamycin antibiotic developed in the 1980s that demonstrates enhanced resistance to beta-lactamases and has a broad spectrum of activity against Gram-negative bacteria. Cefotetan holds potential to be a carbapenem-sparing treatment option. Data on the *in vitro* activity of cefotetan against ESBL-producing *Enterobacterales* remain scarce. Our study assessed the *in vitro* activity of cefotetan against ceftriaxone-nonsusceptible blood culture isolates obtained from patients enrolled in the MERINO trial.

## INTRODUCTION

Among *Enterobacterales*, resistance to third-generation cephalosporins mediated by extended-spectrum beta-lactamase (ESBL) and AmpC beta-lactamase is a major contemporary threat to the health and well-being of individuals globally (1, 2). Approximately 200,000 infections and 9,000 deaths due to ESBL-producing *Enterobacterales* infection in U.S. hospitals occur annually (3). Treatment options for ESBL-producing Gram-negative pathogens are limited compared to those for non-ESBL producers. Indeed, coexisting non-beta-lactamase resistance genes are often identified in these isolates (e.g., *gyrA* and *parC* mutations mediating quinolone resistance in Escherichia coli ST131) (4). Carbapenems have been regarded as the treatment of choice for infection due to ESBL-producing *Enterobacterales* (5). The MERINO trial failed to demonstrate noninferiority, with respect to 30-day all-cause mortality, of piperacillin-tazobactam compared to meropenem for treatment of bloodstream infection due to ceftriaxone-resistant E. coli and Klebsiella pneumoniae (6). Rising use of carbapenems, paired with a rising incidence of carbapenem-resistant organisms globally, has prompted a search for suitable therapeutic alternatives to treat these infections (7).

Cefotetan is a cephamycin antibiotic developed in the 1980s (8). Its unique structure confers enhanced resistance to beta-lactamases and a broad spectrum of activity against Gram-negative bacteria. It is administered via the intravenous and intramuscular routes and has been approved for use in urinary tract, lower respiratory tract, skin and soft tissue, gynecologic, intra-abdominal, and bone and joint infections. Early *in vitro* studies indicated that cefotetan achieved an MIC_90_ of 4 mg/liter against enterobacteria (9). Moreover, a randomized clinical trial of cefotetan versus cefoxitin or moxalactam for treatment of intra-abdominal infection demonstrated superior infection clearance and bacteriologic response with cefotetan (10). Cephamycins, including cefotetan, are unable to be efficiently hydrolyzed by ESBLs and may prove to be a therapeutic alternative to carbapenems. Data on the *in vitro* activity of cefotetan against ESBL-producing *Enterobacterales* remain scarce (11). We aimed to assess the *in vitro* activity of cefotetan against ceftriaxone-nonsusceptible blood culture isolates obtained from patients enrolled in the MERINO trial (6).

## RESULTS

**FIG 1:**
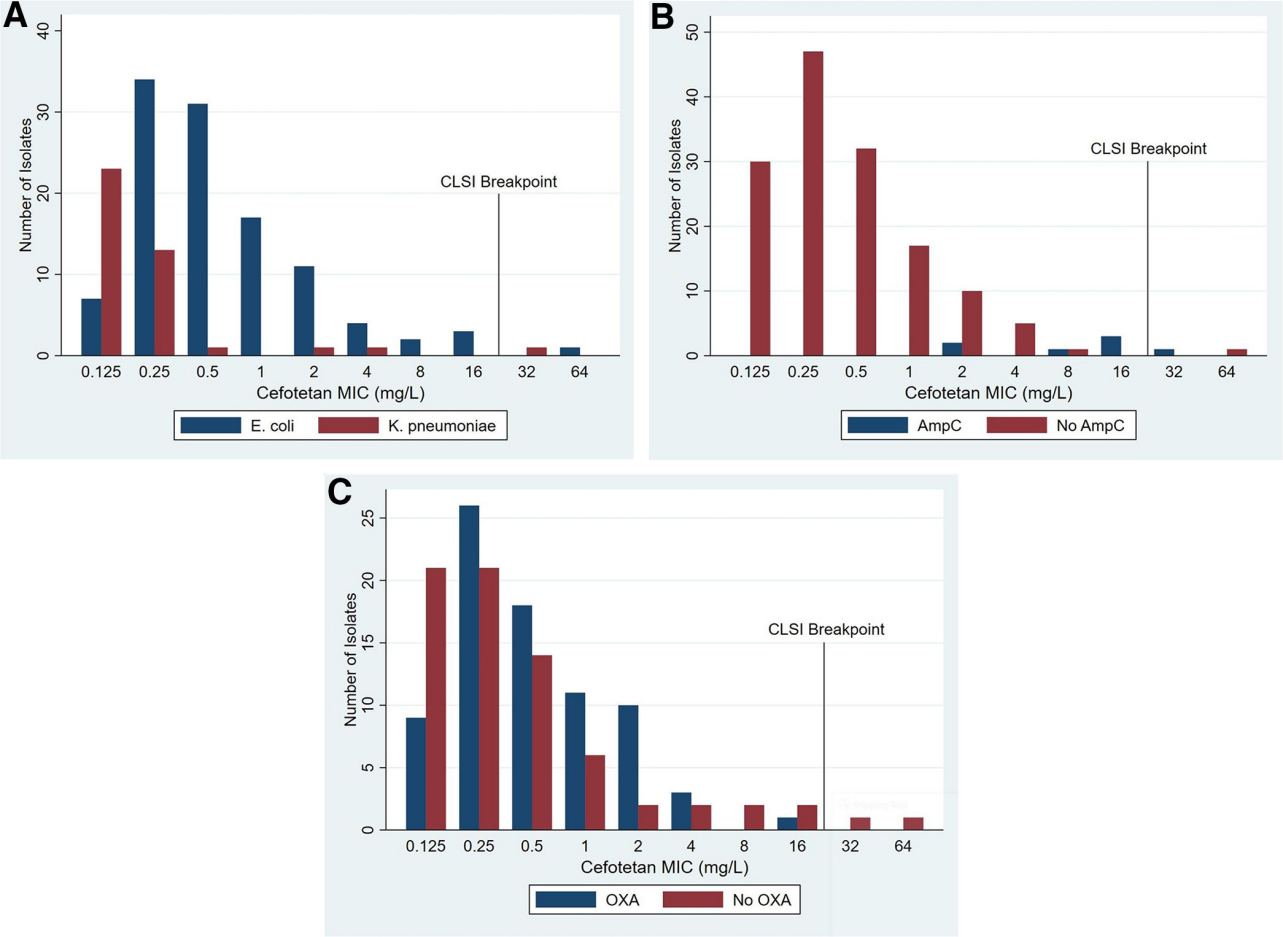
Cefotetan MICs determined by broth microdilution (BMD) of 150 ESBL-producing E. coli and K. pneumoniae isolates by (A) species, (B) AmpC beta-lactamase, and (C) narrow-spectrum OXA beta-lactamase.

One hundred fifty isolates (110 E. coli and 40 K. pneumoniae) from the MERINO trial were collected, and their cefotetan MICs were determined by broth microdilution (BMD). Overall, 98.7% were susceptible according to the CLSI cefotetan susceptible breakpoint, with MIC_50_s and MIC_90_s of 0.25 mg/liter and 2 mg/liter (range, ≤0.125 to 64 mg/liter), respectively. Table 1 presents the cefotetan MIC distribution and percent susceptible according to species and beta-lactamase type. The MIC distributions of E. coli and K. pneumoniae isolates appeared similar, each registering one resistant isolate (64 mg/liter and 32 mg/liter, respectively). The resistant E. coli isolate had *bla*_CTX-M-27_ identified, and the intermediate K. pneumoniae isolate had *bla*_SHV-106_ and *bla*_DHA-1_ present. Overall, MICs appeared higher among isolates with *ampC* genes present, with an MIC_50_ of 16 mg/liter, than among those containing CTX-M-15, which had an MIC_50_ of only 0.5 mg/liter. Indeed, isolates with an *ampC* gene exhibited an overall susceptibility of 85%. Presence of an OXA beta-lactamase did not appear to alter the cefotetan MIC distribution (Fig. 1). The MICs for all the trays testing ATCC strains fell within acceptable ranges. Purity and colony count checks demonstrated pure growth and colony counts ranging from 1 to 9 colonies.

**TABLE 1:** Cefotetan MIC frequency distribution against ESBL-producing E. coli and K. pneumoniae isolates according to species and beta-lactamase type

Organism	No. of isolates with MIC (mg/liter)											% susceptible (CLSI breakpoint)
	≤0.125	0.25	0.5	1	2	4	8	16	32	≥64	Total	
All	30	47	33	16	12	5	3	4	1	1	150	98.7
E. coli	7	34	32	16	11	4	3	4		1	110	99.1
K. pneumoniae	23	13	1		1	1			1		40	97.5
ESBL only (*n* = 67)												
CTX-M-3		4									4	100
CTX-M-14	3	2									5	100
CTX-M-15	14	5	4	3		1	2				29	100
CTX-M-24		1									1	100
CTX-M-27	3	7	7	2						1	20	95
CTX-M-55	1	2	2	1							6	100
CTX-M-134			1								1	100
CTX-M-174						1					1	100
ESBL + OXA (*n* = 74)												
CTX-M-15 + OXA-1	8	24	17	10	9	3					71	100
SHV-12 + OXA-9		1									1	100
CTX-M-15 + OXA-10	1	1									2	100
ESBL + *ampC* (*n* = 7)												
CTX-M-15 + CMY-2								1			1	100
CTX-M-55 + CMY-2							1	1			2	100
CTX-M-15 + CMY-138					1			1			2	100
CTX-M-14 + DHA-1					1						1	100
SHV-106 + DHA-1									1		1	0
ESBL + *ampC* + OXA (*n* = 2)												
CTX-M-15 + CMY-2 + OXA-1								1			1	100
CTX-M-15 + CMY-138 + OXA-1					1						1	100

## DISCUSSION

We demonstrated that almost all ESBL-producing E. coli and K. pneumoniae isolates from our study were susceptible to cefotetan *in vitro*. Unsurprisingly, *ampC*-carrying isolates showed higher MICs overall; *in vitro* resistance to cefoxitin is used as a phenotypic marker to infer the presence of *ampC*, and there exists a structural similarity between cefotetan and cefoxitin. Among AmpC producers, cefoxitin MICs are generally higher than those of cefotetan (12). Isolates harboring the DHA-1 enzyme appeared to have higher cefotetan MICs than those harboring CMY enzymes, although isolate numbers were small. It is unclear whether there is a biological or clinically significant difference in relation to cephalosporinase activity between the two enzyme types.

Isolates producing common CTX-M and narrow-spectrum OXA-type beta-lactamases appeared highly susceptible to cefotetan. Although not a new antimicrobial class, cephamycins have demonstrated promising *in vitro* potency and clinical efficacy against invasive isolates that are resistant to third-generation cephalosporins (13–15). Previous concerns have been put forward over the use of cephamycins for infections with ESBL-producing organisms and development of outer membrane protein (OMP) mutations and/or plasmid-encoding AmpC enzymes during exposure (11). The true significance of this finding from case reports remains uncertain. Cefotetan may be a suitable carbapenem-sparing treatment option for multidrug-resistant *Enterobacterales*, especially those not harboring an *ampC* enzyme. This agent could also be formulated with an inhibitor to mitigate the effect of *ampC*. Cefotetan achieves high plasma levels after intravenous and intramuscular injection and is typically administered twice daily as a 30-min infusion. It achieved a mean plasma concentration of 158 mg/liter at 30 min after a 1-g dose given intravenously to healthy adults. Cefotetan has shown very little *in vitro* activity against Pseudomonas and Acinetobacter species (MIC_90_s, >32 and >32 to 256 mg/liter, respectively) and wide variation in susceptibility against Enterobacter and *Serratia* species (MIC_90_s, 2 to 256 mg/liter and 0.5 to 64 mg/liter, respectively) (8). The lack of activity seen against non-lactose-fermenting Gram-negative organisms may explain why it has not been widely adopted for treatment of urinary tract infection. In the era of emerging multidrug-resistant bacteria, use of pathogen-directed therapies (as opposed to a “cure-all” approach with a single agent) based on species or resistance type may be a useful strategy.

There are a few limitations to this study. Selection of bacterial isolates was restricted to include a subset of nonrandomly selected representative isolates obtained from the MERINO trial. These isolates may not be truly representative of all the resistance mechanisms seen in third-generation-cephalosporin-resistant E. coli and K. pneumoniae globally. Antimicrobial susceptibility testing was performed using an automated digital antibiotic dispensing platform (Tecan D300e; Tecan Trading AG, Switzerland). In precision studies assessing the performance of this platform in *Enterobacteriaceae*, essential and categorial agreement levels were 96.8% and 98.3%, respectively (16). This finding supports the accuracy of this approach for use in BMD testing. The clinical efficacy of cefotetan for infection due to ESBL producers remains uncertain but warrants further study.

### Conclusion.

Cefotetan demonstrated favorable *in vitro* efficacy against ESBL-producing E. coli and K. pneumoniae bloodstream isolates with MIC_50_s and MIC_90_s of 0.25 mg/liter and 2 mg/liter (range, ≤0.125 to 64 mg/liter), respectively. Higher MICs were seen in isolates coharboring an *ampC* beta-lactamase. Cefotetan may have a place for therapeutic use as a carbapenem-sparing therapy for infection due to these organisms.

## MATERIALS AND METHODS

### Bacterial isolates.

The MERINO trial recruited patients with bloodstream infections due to third-generation-cephalosporin-nonsusceptible E. coli and K. pneumoniae in nine countries from February 2014 to July 2017 (6). All blood culture isolates from enrolled patients were stored and had previously undergone whole-genome sequencing (WGS) to detect antimicrobial resistance genes. A subset of isolates that had at least one ESBL gene identified were chosen to be included in this study. Ultimately, isolates containing different combinations of CTX-M ESBLs, narrow-spectrum OXA and SHV enzymes, and AmpC beta-lactamases were used. Each isolate was subjected to broth microdilution (BMD) testing for cefotetan MIC determination.

### Antibiotic preparation.

Cefotetan powder (Glentham Life Sciences, GA5476) was dissolved in DMSO (Thermo Fisher, D/4121/PB08) at a concentration of 10,000 mg/liter. This stock solution was loaded directly to the Tecan D300e (Tecan Trading AG, Switzerland) T8 print cartridge.

### Broth microdilution tray preparation.

The concentration range (0.125 to 64 mg/liter) were chosen to include both recommended reference strains, CLSI breakpoints (susceptible, ≤16 mg/liter; intermediate, 32 mg/liter; resistant, ≥64 mg/liter) (see Table 2A in reference 17), and attainable therapeutic concentration (128 mg/liter). The tray layout was designed in Tecan D300e Control software. Prepared antibiotic was dispensed into labeled 96-well trays (Thermo Scientific, 262162) which were inoculated within 20 min of printing.

### Quality control.

Two ATCC strains were used to check the performance of each batch of trays: Escherichia coli ATCC 25922 (target MIC, 0.125 mg/liter) and Staphylococcus aureus ATCC 29213 (target MIC, 8 mg/liter) (see Table 5A-1 in reference 17). A separate tray was prepared to check E. coli ATCC 25922 at lower concentrations, ranging from 0.004 to 2 mg/liter.

### Isolate preparation.

Test and reference isolates were stored in brain heart infusion (BHI) broth (BD, Bacto 237500) containing 30% glycerol (Chem-Supply, GA010) at −80°C. A scraping from the frozen vials was streaked onto 5% Columbia horse blood agar (HBA) (Edwards, MM1085) and incubated at 37°C in ambient atmosphere for 18 to 24 h. A single colony of each was subcultured to fresh HBA and incubated under the same conditions. Two or three colonies of each isolate were collected using a sterile rayon swab and resuspended in sterile normal saline (0.9% NaCl; Chem-Supply, US008779). Turbidity was adjusted to a 0.5 McFarland standard as read using DensiCHEK Plus (bioMérieux, France). Five microliters of inoculated saline was added to 1 ml of cation-adjusted Mueller-Hinton broth (CAMHB) (BD, BBL 211322) and vortexed, to achieve an approximate concentration of 5 × 10^5^ CFU/ml. Fifty microliters of inoculated broth was dispensed into each into each well of a single row on the BMD tray using an electronic repeat-dispense pipette. Purity and colony count checks were performed by collecting a 1-μl loop of broth from the positive-control well for each isolate and streaking onto half of an HBA plate. A second 1-μl sample from the same well was diluted in 100 μl of sterile saline, and 1 μl was streaked on the other half of the plate. Plates showing pure growth on the undiluted streak and 1 to 10 colonies on the diluted streak passed purity and colony count checks.
